# Understanding the impacts of temperature and precipitation on antimicrobial resistance in wastewater: theory, modeling, observation, and limitations

**DOI:** 10.1128/msphere.00947-24

**Published:** 2025-03-05

**Authors:** Carly Ching, Indorica Sutradhar, Muhammad H. Zaman

**Affiliations:** 1 Department of Biomedical Engineering, Boston University, Boston, Massachusetts, USA; 2 Center on Forced Displacement, Boston University, Boston, Massachusetts, USA; University of Michigan, Ann Arbor, Michigan, USA

**Keywords:** wastewater, antibiotic resistance, climate

## Abstract

Changing climate may contribute to increased antimicrobial resistance (AMR), particularly in wastewater which acts as a reservoir for resistant bacteria. Here, we determined how applying climate dependencies to our previously published model, rooted in theory, impacts computational simulations of AMR in wastewater. We found AMR levels were reduced at lower temperatures but increased with lower precipitation. The impact of precipitation on AMR was more pronounced at higher temperatures compared to lower temperatures. To validate our model, we investigated associations between total AMR gene abundance in wastewater from the Global Sewage Surveillance project and mean temperature and rainfall values extracted from European Centre for Medium-Range Weather Forcasts Reanalysis v5 (ERA5) reanalysis. We observed similar trends between the simulations and observations. Observations and simulations from our study can inform experiments to determine causal relationships as well as help identify other key drivers. We also discuss study challenges given the complex nature of AMR in the environment.

## INTRODUCTION

Climate change is a pressing threat to human health and well-being through the amplification of current health threats (1). Studies have shown that climate change can cause increases in infection and respiratory distress, among others (2). Climate change also has the potential to cause emerging threats to human health, including the development of multi-drug-resistant pathogens (3). There is growing concern that climate change will increase bacterial antimicrobial resistance (AMR) (4), which renders clinicians unable to treat bacterial infections. Underlying this concern are the mechanisms by which climate stress can lead to physiological responses in bacteria that promote AMR development (4). Furthermore, extreme climate events can alter infrastructure and human behavior, which can also promote AMR development and spread.

The environmental dimension of AMR is increasingly becoming paramount (5). In the environment, ambient temperature and precipitation may have a more direct impact on bacterial numbers and processes compared to within a host. One especially important environmental reservoir is wastewater, which is an environment in which waste containing bacteria and AMR genes can persist (6). Wastewater (i.e., industrial and agricultural wastewater and domestic wastewater also known as sewage) surveillance data, in which samples are collected and markers for antibiotic resistance genes are detected using metagenomic sequencing methods, can serve as a data proxy for measuring AMR burden in the environment (7). Global analyses of untreated sewage have identified 1,625 different AMR genes and shown that gene abundance varies by region. The most abundant AMR genes encoded resistance to macrolides, tetracyclines, aminoglycosides, beta-lactams, and sulfonamides (7). Furthermore, it has been suggested that wastewater surveillance better captures data in low- and middle-income countries (LMICs) where clinical surveillance may be more challenging due to limited resources. For example, a study from Bangladesh found that severe acute respiratory syndrome coronavirus 2 (SARS-CoV-2) was consistently detected in wastewater even though there were large differences in reported clinical cases or periods with no cases (8). However, it should be noted that due to potential differences in the viability of viruses (9, 10) compared to bacteria (which may add dynamics to resistance), there may be discrepancies in how accurately surveillance data reflect clinical surveillance.

Existing analyses of the climate-AMR nexus typically involve the integration of climate and AMR data to determine any significant associations or correlations, given that it is challenging to generate direct evidence for causation. This is because AMR has multiple drivers, of which climate is one (11, 12). Regional studies have found that antibiotic resistance increases with temperature in the United States, Europe, and China (13–15), and more recent global analyses have also found a correlation to increased temperature (12, 16). Notably, these studies utilize clinical surveillance data, where human burden, transmission, and reporting may be more influenced by infrastructure and access to healthcare, leading to bias (12). As such, it is an aspiration to move beyond correlational studies with mechanistic studies or controlled experiments. One important tool to investigate the impact of climate on AMR is computational modeling, which allows for simulations that are difficult to measure in the environment.

Currently, there is limited understanding of the dynamics of how climate impacts AMR in wastewater. Here, we test the hypothesis that temperature and precipitation can alter the abundance of AMR genes in wastewater. Based on the literature, we first modeled the impact of key climate parameters on resistance development. We then compared the model outputs to observations from published wastewater surveillance data and public climate data. We also open the discussion and conversation about the value and limitations of correlative studies in the landscape of the complex problem of AMR. This work can help us further research and better understand how climate change drives AMR in aquatic environmental reservoirs.

## MATERIALS AND METHODS

### Computational modeling

In this paper, we modified a computational model based on a previously developed model of the growth of antibiotic-resistant bacterial populations in continuous flow environments (17). This model consisted of ordinary differential equations representing antibiotic-susceptible and antibiotic-resistant bacterial populations, with inputs including the growth rates of antibiotic-susceptible and -resistant strains and mutation rates in response to subinhibitory antibiotics. The model was based on studies of AMR development *in vitro* and in agricultural sludge and validated through experimental studies modulating antibiotic concentrations using the eVOLVER system (17). In this work, we expanded the model beyond the effect of only environmental antibiotic residues to study the additional impact of changes in temperature and rainfall on AMR development. These environmental factors were modeled as parameters temp and rain. Temperature was modeled as parameter temp as the fraction of growth rate at a given temperature compared to ideal growth (37°C). Thus, high temperature was modeled at ~25°C as temp *=* 0.25 and low temperature was modeled at ~16°C as temp = 0.1 based on published growth rates (18). High rainfall was modeled as parameter rain with high rainfall modeled as a 1% increase in inflow and outflow (rain = 1.01). These parameters, alongside the previously modeled subinhibitory antibiotic concentration (50% inhibitory concentration), were then incorporated into the terms for flow rate, induced mutation to AMR, AMR gene transfer, and bacterial growth based on general temperature dependencies found in previous literature, namely that decreases in temperature decrease mutation rate and gene transfer (18–22) and increased precipitation would lead to lower bacteria concentrations and subsequently lower gene transfer (23, 24). We note that we included simplified temperature dependencies, and there is not always a defined 1:1 relationship. The temperature and climate dependencies used are summarized in Table 1.

**TABLE 1:** Temperature and precipitation dependencies in the AMR model

	Temperature	Precipitation
Inflow and outflow rate	No dependency	rain∗flow rate
Induced mutation rate	temp∗mutation rate	No dependency
Gene transfer rate	$-0.1*\left[{\left(temp-1\right)}^{2}+1\right]*gene\;transfer$	$\frac{1}{rain}*gene\;transfer$
Growth rate	temp∗growth rate	No dependency

### Data integration

Total fragments per kilobase of transcript per million mapped reads (FPKM) of antibiotic resistance genes (total AMR abundance), encompassing 408 gene groups, covering 14 antibiotic classes) identified using metagenomics from previously published wastewater (untreated sewage) data by the Global Sewage Surveillance project (GSSP) (7) were extracted and integrated with the average European Centre for Medium-Range Weather Forcasts Reanalysis v5 (ERA5) reanalysis (25) of monthly mean temperature (°C), 2 m above the surface and monthly mean of total rainfall per day ([mm/day]) from 1997 to 2016. Quantitative data are provided in Table S1. Further stratified analyses were performed with other variables extracted, including World Bank income classification, main Köppen climate classification group (26) (A [tropical], B [arid], C [temperate], D [continental], E [polar]), and region. Spearman correlation was performed as the data did not pass a normality test. Extracted data are provided in Table S1, and expanded methodology is provided in the supplemental material.

## RESULTS

### Interaction of temperature and precipitation

The main relationship found in current published work on regional and global levels using clinical surveillance is that AMR increases with temperature (13–15). This increased AMR can theoretically be attributed to the growth advantage that some bacteria have (increased growth rate) at higher temperatures (27), which allows for increased evolution. Evidence also suggests that warmer temperatures increase the number of mutations with beneficial fitness effects and magnitude of positive selection (28). Warmer temperatures are also associated with increases in induced mutation rate (18) and offsetting fitness costs associated with antibiotic resistance (selection) (4, 28–30). Furthermore, gene transfer between different bacterial species has been shown to increase with temperature, reaching an optimal temperature between 24.9°C and 29°C, depending on nutrient differences (22), although the relationship can vary (21). However, it has also been shown that increased precipitation leads to reduced numbers of bacteria in wastewater (24), and that bacterial concentration impacts gene transfer (23). Thus, there are two theoretical counteracting effects occurring that impact bacteria within wastewater; increased temperature increases induced mutations and fitness, while increased precipitation could reduce the total concentration of bacteria available for gene transfer or quorum sensing, as well as dilute substances that drive AMR development, such as antibiotic residues.

### Climate-dependent computational modeling

**Fig 1:**
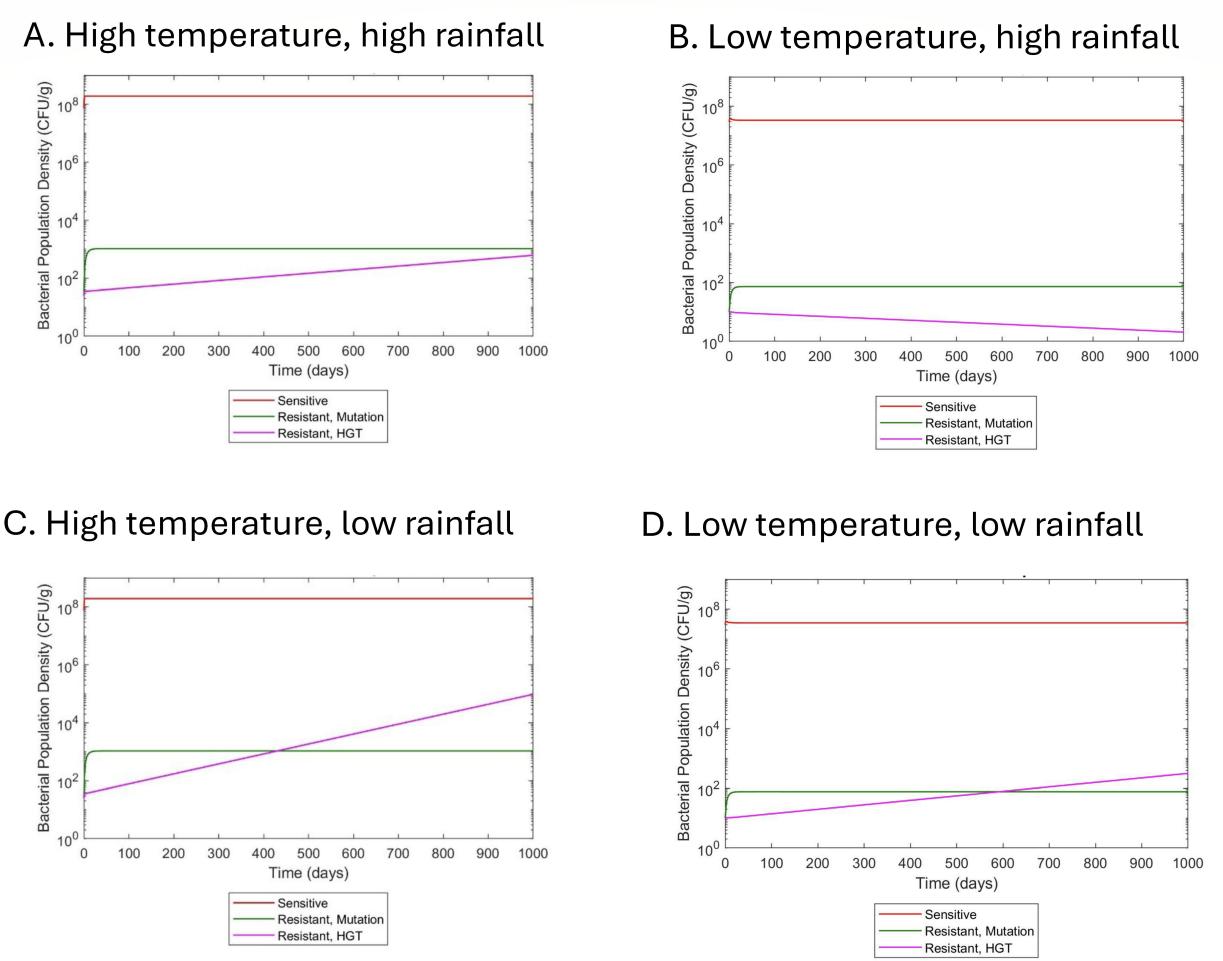
Computation simulations of antibiotic-resistant bacterial population development over time at various temperatures and flow rates. (**A**) Temperature = 25°C and flow = 1.01; (**B**) temperature = 15°C and flow = 1.01; (**C**) temperature = 25°C and flow = 1; (**D**) temperature = 15°C and flow = 1.

**Fig 2:**
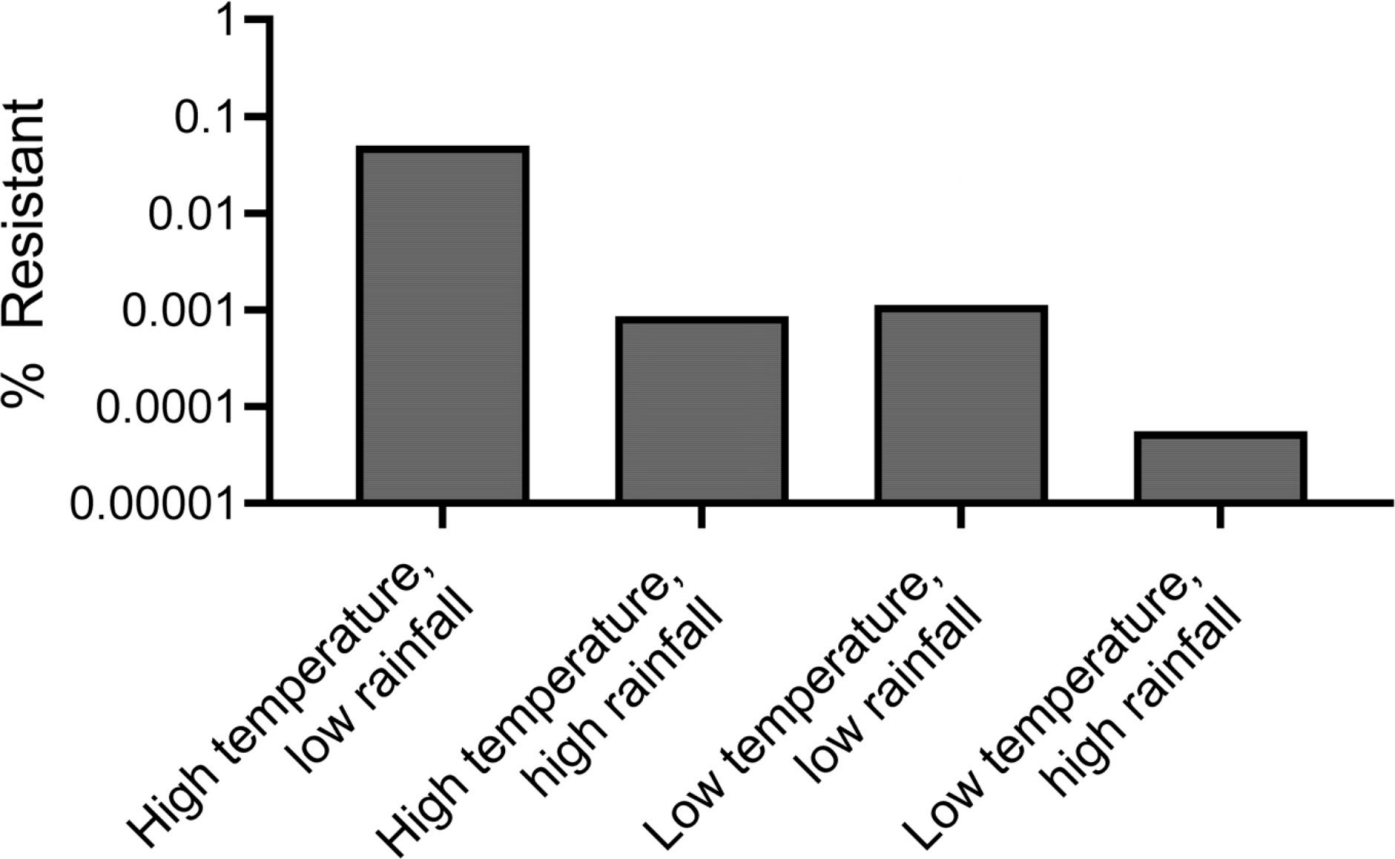
Percent resistant out of the total number of bacteria for various climate scenarios simulated with the computational wastewater model.

Based on the above hypothesized interaction of two key climatic factors, temperature and precipitation, we added temperature and precipitation dependencies to our previous model. The previous model for wastewater, based on reference 17, is amenable to wastewater because it models resistance in continuous flow conditions and includes parameters for induced mutation rate and gene transfer (17). Based on trends from previous literature (18–24), we simulated high temperature (~25°C) and low temperature (~16°C) scenarios with either high or low levels of rain. We observed that after the computational simulations have been run, the highest ratio of resistant bacteria to sensitive bacteria is in the high temperature, low rainfall scenario, followed by very similar values in the high temperature, high rainfall and low temperature, low rainfall scenarios (Fig. 1 and 2). The low temperature, high rainfall scenario has the lowest AMR load. We also found that precipitation has less impact on AMR abundance at low temperatures compared to high temperatures (3× more of a decrease between high and low precipitation at 25°C) (Fig. 2). This suggests a balance between temperature and precipitation on bacterial processes and growth, and bacterial concentrations in environmental water reservoirs. Additionally, sensitivity analysis of the model demonstrated that the model had higher sensitivity to a 5% perturbation in rainfall than a 5% perturbation in temperature or a 5% perturbation in environmental antibiotic levels (Fig. S1).

### AMR gene abundance in wastewater is positively associated with temperature globally

**Fig 3:**
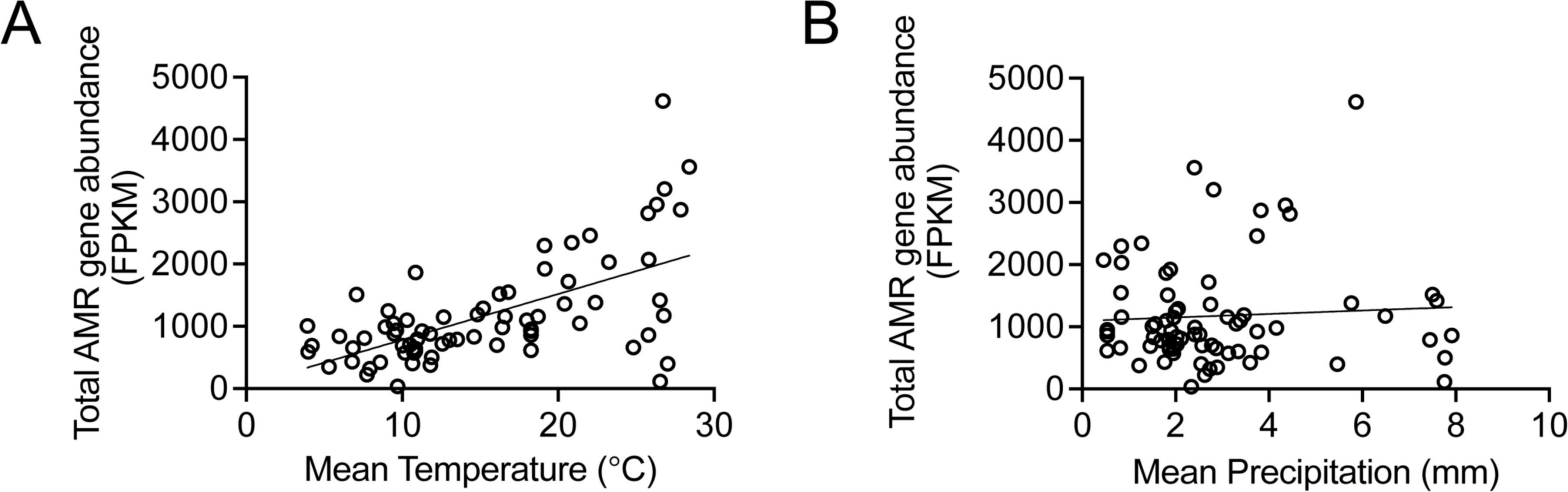
(A) Total AMR FPKM plotted against the mean annual temperature from 1997 to 2016. Region. Spearman correlation, *r* = 0.5529, *P* < 0.0001 (73 pairs). (B) Total AMR FPKM plotted against mean annual precipitation per day from 1997 to 2016. Spearman correlation, *r* = −0.006 (73 pairs), NS (*P* = 0.9587). Temperature and precipitation data are from ERA5 reanalysis (25) and AMR data are from GSSP (7). Line represents simple linear regression.

To complement and validate our computational simulations, we looked to available data sets. The GSSP represents one of the largest data sets of wastewater sampling and testing for AMR, unique in that sampling is comparable across many sites (7). Integrating mean temperature and wastewater AMR surveillance data, on a global level, we observed a significant association (Spearman correlation, *r* = 0.6020, *P* < 0.0001) between total standardized AMR gene abundance and mean temperature (average monthly, over 20 years) (Fig. 3A), but no significant association (*r* = −0.006, *P* = 0.9587) between total resistance gene abundance and mean precipitation (Fig. 3B). This suggests that temperature influences abundance of AMR within wastewater, and specifically that warmer environments are correlated with increased resistance levels, which is supported by published work using clinical surveillance (13–15). However, Hendrickson et al. did not find an association between total AMR gene abundance and temperature on the day of sampling (7), suggesting the impact of temperature may be on a longer timescale. Notably, we observed that at higher temperatures above 25°C, close to the optimal environmental temperature for growth (31) and gene transfer (22), the relationship to AMR appears to break down (Spearman correlation between sites with mean temperatures above 25°C and total AMR gene abundance has a *P*-value = 0.36, standard deviation = 1,387), suggesting a counteracting factor such as precipitation.

### High levels of precipitation associated with decreased AMR gene abundance in wastewater in tropical climates

**Fig 4:**
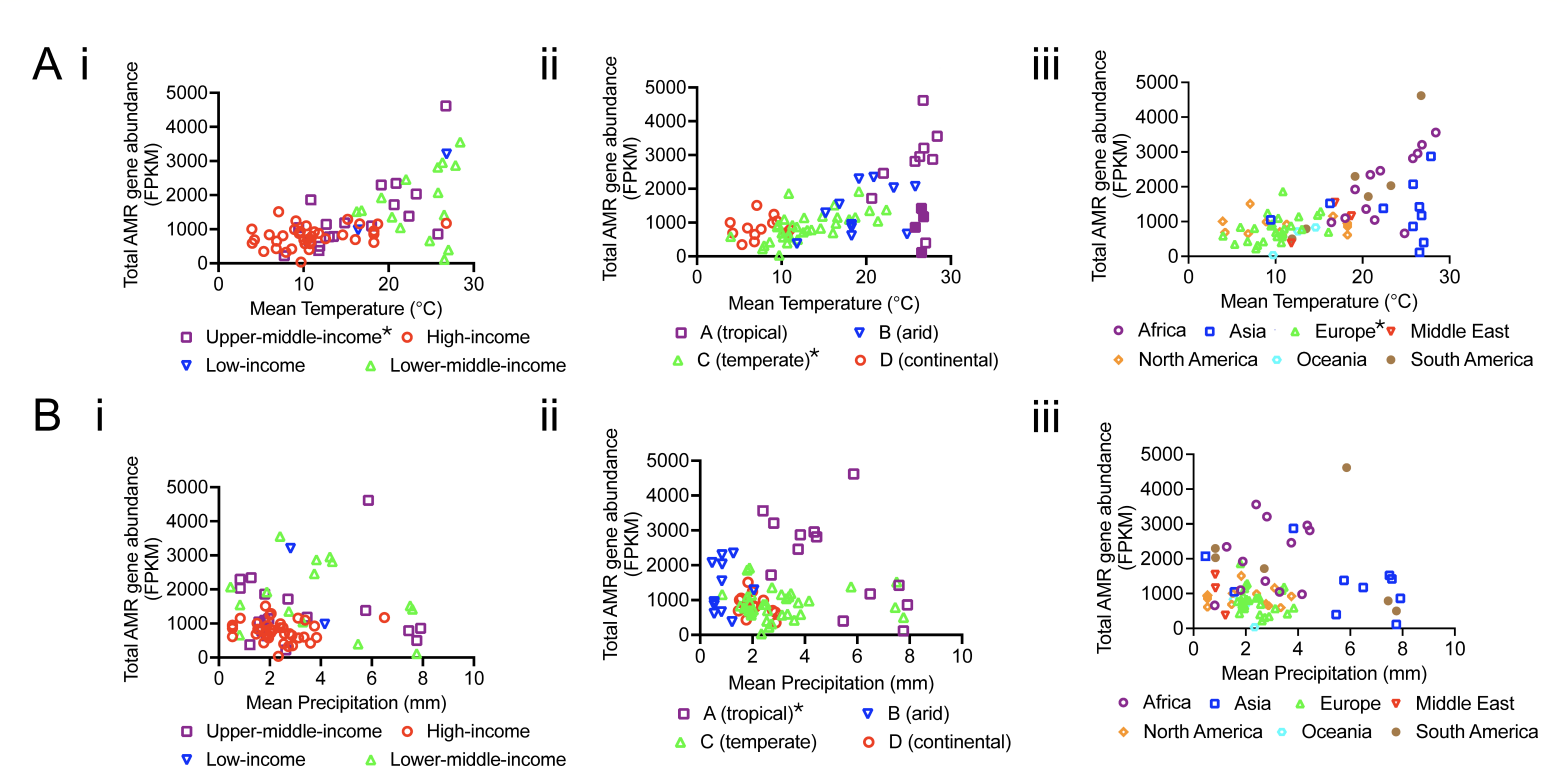
(A) Total AMR FPKM plotted against the mean annual temperature from 1997 to 2016, stratified by (i) World Bank income classification, (ii) Köppen climate group, and (iii) region. Filled boxes in Aii indicate tropical countries with average precipitation over 6.5 mm. (B) Total AMR FPKM plotted against mean annual precipitation per day from 1997 to 2016, stratified by (i) World Bank income classification, (ii) Köppen climate group, and (iii) region. Each sample is represented by one dot. Temperature and precipitation data are from ERA5 reanalysis (25) and AMR data are from GSSP (7). Spearman correlation was performed on each stratified data set, and significant associations are indicated as a star on the corresponding legend.

**Fig 5:**
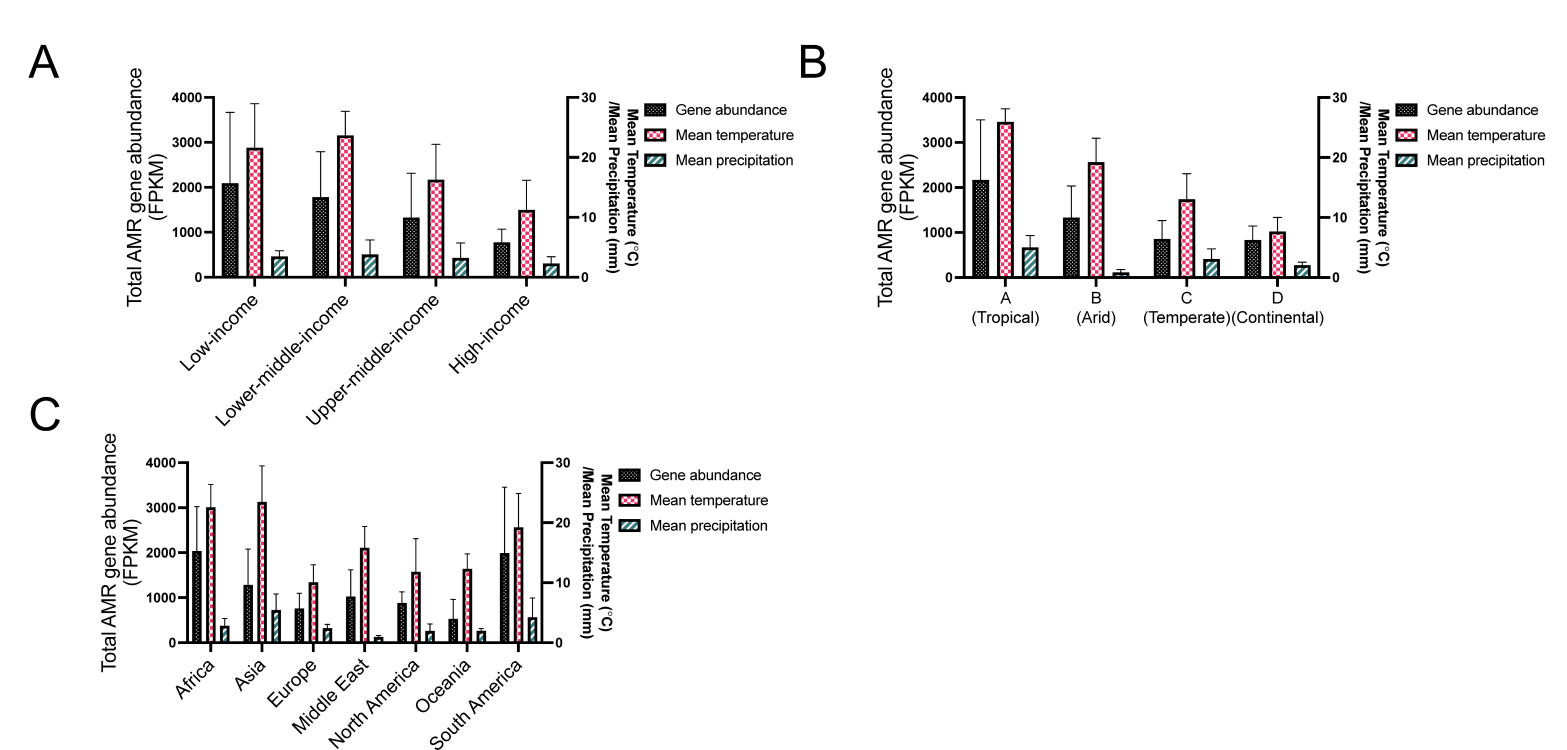
Mean total AMR FPKM, mean annual temperature from 1997 to 2016, and mean annual precipitation per day from 1997 to 2016 stratified by (A) World Bank income classification, (B) Köppen climate group, and (C) region. Error bars represent standard deviation. Temperature and precipitation data are from ERA5 reanalysis (25), and AMR data are from GSSP (7).

Stratifying the data in Fig. 4 by climate, country economy, and region can help identify specific relationships or the breakdown of global relationships. As expected, climate clusters to different ranges of mean temperature, which also overlaps with country economy and region (Fig. 4 and 5). However, once stratified, only upper- to middle-income countries (*r* = 0.6677, *P* ~ 0.0025, 18 pairs), European countries (*r* = 0.4817, *P* = 0.0127, 26 pairs), and temperate climate (*r* = 0.7149, *P* < 0.001, 34 pairs) countries had significant associations to temperature, although there was too small a sample size for many of the correlations to be performed (Fig. 4). Observing the breakdown of the relationship of AMR gene abundance to temperature after 25°C, we found that these sites were mainly in tropical climates, which also have more precipitation on average (Fig. 4Aii and 5B). Moreover, precipitation in tropical climates had the only significant negative association with AMR abundance; there was a significant negative association between mean precipitation and AMR abundance in tropical climates (*r* = −0.6154, *P* = 0.0284, 13 pairs) (Fig. 4Bii), and the tropical countries with average precipitation over 6.5 mm correspond with lower AMR gene abundances (filled boxes, Fig. 4Aii). Tropical countries are also mainly in Africa and Asia, with Asian countries having higher mean precipitation (Fig. 4Biii and 5C). Interestingly, Asian countries had a lower AMR abundance (Fig. 4Biii and 5C), suggesting that heavy precipitation decreases AMR levels in wastewater. This further suggests an interplay between temperature and precipitation may mediate resistance reservoirs. As with the computational simulations, we also observe that at ~25°C and above in the data, there are sites with high rainfall that have similar AMR gene abundance to sites with low temperatures (Fig. 2 and 4Aii).

### Seasonality may impact wastewater AMR load

While there is a global relationship between temperature and AMR abundance, there are overlapping variables. Hendriksen et al. found that irrespective of AMR gene diversity, the total AMR abundance was highly correlated with certain World Bank variables, mainly concerning sanitation and health (7). Comparing the average AMR gene abundance between country economies (World Bank income classification), climates (Köppen climate classification group), and regions, we observe that for country economy, high-income countries had the lowest AMR gene abundance and smallest standard deviation (black bars, Fig. 5A), while also having the lowest mean temperature (pink bars, Fig. 5A).

Climate is extrinsically linked to temperature and precipitation, but also represents other variables such as seasonality (Fig. 5B). It has also been shown that bigger spreads in temperature may impact the fitness of antibiotic-resistant bacteria more, as decreased thermal niche breadth has been shown to be a trade-off for antibiotic resistance (30). Thus, as climate provides information about seasonality, continental climates which have greater temperature extremes might lead to increased cycles of time when antibiotic-resistant bacteria endure greater fitness costs and lower growth compared to antibiotic-sensitive bacteria, in addition to lower growth overall (30). Indeed, continental climates (Fig. 5B) had the lowest total AMR gene abundance, followed by temperate climates, which also have seasonal differences and cold winters but milder temperature extremes. There was a bigger deviation in AMR abundance in arid and tropical climates (Fig. 5B), which experience temperatures closer to optimal growth and warm and dry winters.

## DISCUSSION AND LIMITATIONS

Overall, we present a hypothesis that there are counteracting effects between elevated temperature and elevated precipitation on AMR in wastewater, supported by theory, modeling, and observation. Notably, these analyses suggest that regional differences and differences between different economies in AMR may in part be associated with differences in climate. This is also interesting because the discourse around LMICs having higher burdens of AMR is often centered around differences in antibiotic consumption and socioeconomic behaviors, without discussion of climate (32, 33), which is an intrinsic difference. However, we cannot infer causation from our analyses and increased AMR gene abundance in wastewater does not directly inform on transmission. Thus, while our work presents an intriguing hypothesis, further evidence is needed, and we call for expanded longitudinal work with bigger sample sizes, which will enable more robust analyses. Our observations can help inform hypotheses for controlled field and laboratory experiments. We also find that the relationship between climate and AMR in the environment may not be explained by a singular association; by observing global data, we can identify specific trends and populations that are more vulnerable. Observations from correlative studies can also identify outliers and subsequently other key drivers, such as wastewater composition. Moreover, care needs to be taken with global analyses, as we see that associations often break down once stratified (Fig. 4).

The environment and climate are two emerging dimensions of AMR. One current limitation is the lack of standardized methods for wastewater surveillance, as opposed to the more established clinical surveillance techniques, which hinders the comparability of findings across different studies. Integrating clinical and environmental surveillance will allow a more comprehensive insight into global AMR trends (34). Another hurdle in investigating the causal role of climate and AMR is navigating confounding variables, such as socioeconomic status, public health policies, and antibiotic consumption. From a One Health perspective, additional confounders also include water pollution and antibiotic use in agriculture and poultry, and confounders can get more granular such as population age distribution and regional differences in AMR management. Thus, given the many drivers and associated factors of AMR, is it even possible to account for all confounding variables? The answer is practically no. In this situation, we open the conversation to what are responsible ways to use the data, what standards should be used, and how can we use the data to support hypothesis-driven work given the challenges in generating direct evidence for causation. Another weakness is an overreliance on statistical significance to deem correlation studies acceptable or informative. For example, with a big enough sample size, a weak correlation can be statistically significant, but that does not mean findings are clinically significant (35). It should also be noted that confounder is a causal concept (36), and causation is explicitly not inferred within studies that integrate data sets. Furthermore, our study only looked at two climatic factors. However, other environmental variables like altitude and pressure can also impact bacterial physiology (37). Ultimately, efforts to reduce climate change and increase preparedness for outbreaks in the event of extreme weather events, even without causal inference due to multiple drivers, can help reduce the burden of infectious disease.

## Data Availability

Data used for this study is either publicly available or available on request.

## SUPPLEMENTAL MATERIAL

Supplemental material msphere.00947-24-s0001.pdf Expanded methodology and Fig. S1.

Table S1 msphere.00947-24-s0002.xlsx Extracted AMR gene abundance and climate data for each sampling site.

ASM does not own the copyrights to Supplemental Material that may be linked to, or accessed through, an article. The authors have granted ASM a non-exclusive, world-wide license to publish the Supplemental Material files. Please contact the corresponding author directly for reuse.
