# Supplementary material for: Understanding the impacts of temperature and precipitation on antimicrobial resistance in wastewater: theory, modeling, observation, and limitations
Source: mSphere. 2025 Mar 5;10(3):e00947-24. doi: 10.1128/msphere.00947-24 (PMC11934317; doi:10.1128/msphere.00947-24)
Supplement: Supplemental material — Expanded methodology and Fig. S1. [file msphere.00947-24-s0001.pdf]

**Supplemental Annex 1**

**Data integration**

Total fragments per kilobase of transcript per million mapped reads (FPKM) of antibiotic resistance genes (total AMR abundance) identified using metagenomics from previously published wastewater data by the Global Sewage Surveillance project (1) (was extracted. Sampling occurred in early 2016 and each sampling site provided longitude and latitude coordinates. Extracted data is provided in Table S1. Using European Centre for Medium-Range Weather Forcasts Reanalysis v5 (ERA5 reanalysis) (2) the monthly mean temperature [°C], 2m above the surface and monthly mean of total rainfall per day [mm/day]) for all months from 1997-2016 was extracted. ERA5 reanalysis (2) was retrieved using the API code from the EU Copernicus Climate Data Store (https://cds.climate.copernicus.eu/), altered to retrieve the corresponding longitude and latitude of each sampling site, with ,nc files read using NASA Panoply netCDF, HDF and GRIB Data Viewer (https://www.giss.nasa.gov/tools/panoply/). Raw data was extracted to an excel spreadsheet and the average across all years was calculated. For mean temperature and rainfall, the longitude and latitude were used for each site except a subset lacking data in which longitude and latitude within 30km was used (indicated in Table S1). Further stratified analyses were performed with other variables extracted including World Bank income classification, main Köppen climate classification group (3) (A (tropical), B (arid), C (temperate), D (continental), E (polar)) and region. Data was plotted against each other, and a Spearman correlation was performed as the data did not pass a normality test.

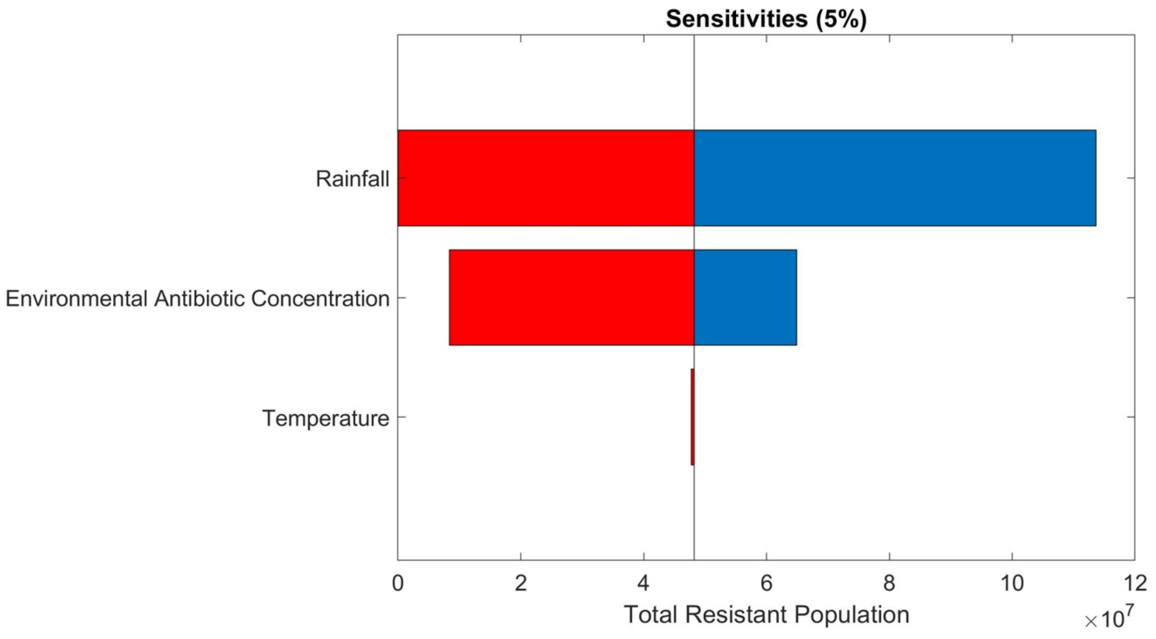


**Figure S1.** Sensitivity analysis of rainfall, temperature, and environmental antibiotic concentration for model of antibiotic resistance in wastewater. Rainfall (*rain),* temperature (*temp*) and environmental antibiotic concentration (*E)* input parameters were modulated ± 5% from a baseline of 1 and output of total resistant bacterial population (sum of bacteria resistant from mutation and bacteria resistant from horizontal gene transfer) was plotted with respect to the baseline total resistant bacterial population.

**References:**


1. Hendriksen RS, Munk P, Njage P, van Bunnik B, McNally L, Lukjancenko O, Röder T, Nieuwenhuijse D, Pedersen SK, Kjeldgaard J, et al. 2019. Global monitoring of antimicrobial resistance based on metagenomics analyses of urban sewage. Nat Commun 10:1124. https://doi.org/10.1038/s41467-019-08853-3

2. Hersbach H, Bell B, Berrisford P, Hirahara S, Horányi A, Muñoz-Sabater J, Nicolas J, Peubey C, Radu R, Schepers D, et al. 2020. The ERA5 global reanalysis. Quart J Royal Meteoro Soc 146:1999–2049. https://doi.org/10.1002/qj.3803

3. Beck HE, Zimmermann NE, McVicar TR, Vergopolan N, Berg A, Wood EF. 2018. Present and future Köppen-Geiger climate classification maps at 1-km resolution. Sci Data 5:180214. https://doi.org/10.1038/sdata.2018.214
